# Pulsating enophthalmos in association with an orbital varix

**DOI:** 10.4103/0301-4738.49399

**Published:** 2009

**Authors:** Venkatesh C Prabhakaran, Dinesh Selva

**Affiliations:** Oculoplastic and Orbital Division, Department of Ophthalmology and Visual Sciences, University of Adelaide and the South Australian Institute of Ophthalmology, Adelaide, Australia

**Keywords:** Orbital varix, pulsating enophthalmos

## Abstract

We report a case of pulsating enophthalmos secondary to orbital varix associated with orbital bony defects. A 64-year-old female with pulsating enophthalmos of the right eye was found to have a right orbital mass with bony defects of the orbit. Valsalva maneuver failed to induce proptosis. The diagnosis of orbital varix was confirmed by exploratory orbitotomy. During general anesthesia for orbitotomy, proptosis of the right eye was noted. Ophthalmologists should be aware of the association between orbital varices and cranial bony defects and encephaloceles. Proptosis induced by general anesthesia and positive pressure ventilation suggests an underlying distensible venous anomaly.

Orbital varices are vascular hamartomas consisting of a plexus of low-pressure, low-flow, thin-walled vessels that communicate with normal orbital veins.[1,2] They usually present with proptosis that may increase with the Valsalva maneuver if the malformation is distensible. Less commonly, they may be associated with enophthalmos. Recently, defects of the cranium and facial skeleton have been reported in association with orbital varices.[2] We report a case of orbital varix with pulsating enophthalmos secondary to bony defects in the orbital wall.

## Case Report

A 64-year-old Caucasian woman was referred for an incidentally discovered right orbital mass on computed tomographic (CT) scan. Her ocular history was significant for bilateral trabeculectomies performed for pseudoexfoliative glaucoma. She had noticed that her right eye was sunken-in since childhood but attributed it to a blunt injury to the face that she had suffered as a child. Pulsation of her right eye had been noticed during previous ophthalmic examination.

On examination, her visual acuity was 20/100 right eye and 20/60 left eye. Her right eye was enophthalmic (Hertel's exophthalmometer readings: 13mm right eye, 17mm right eye with 110mm base) [Fig. 1a]. Pulsations of the right eye, coincident with the arterial pulse, were obvious when viewed from the side. Ocular examination revealed filtering blebs in both eyes and significant glaucomatous optic atrophy of both optic discs. Positional change and Valsalva maneuver did not induce proptosis.

**Figure 1a:**
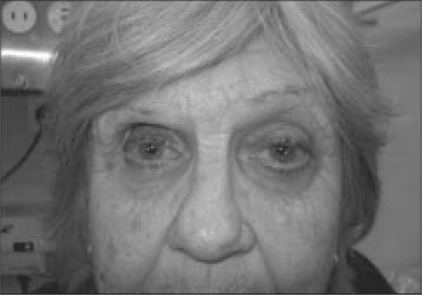
Clinical photograph demonstrating enophthalmos of the left eye

**Figure 1b:**
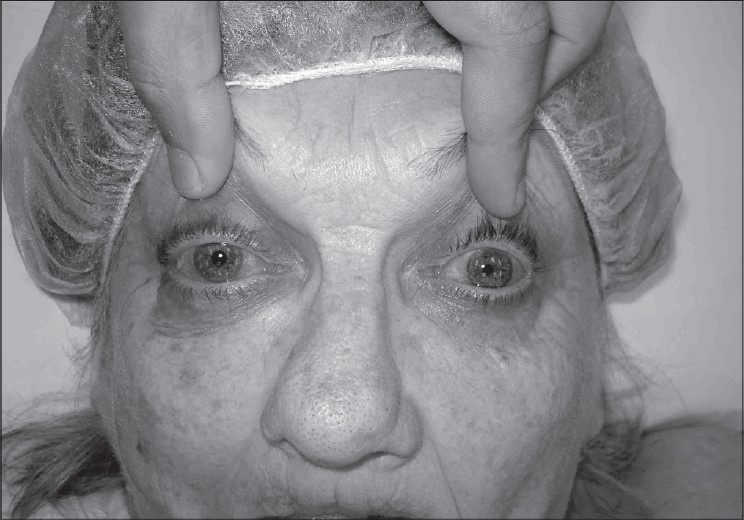
Intraoperative photograph showing right eye proptosis that developed upon induction of general anesthesia

**Figure 1c:**
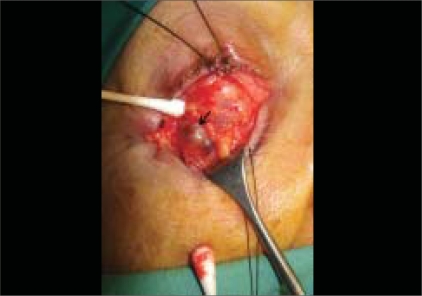
Intraoperative photograph demonstrating the thin distensible vascular channels characteristic of an orbital varix (arrow)

Computed tomographic scan showed a diffuse, moderately enhancing mass in the right inferior orbit. Large bony defects were noted in the medial wall and greater wing of sphenoid on the right side [Fig. 2a]. There was no change in the size of the orbital mass between axial and coronal scans.[3] Magnetic resonance imaging (MRI) scans revealed an intensely enhancing, irregular mass filling the right inferior orbit, with flow voids [Fig. 2b]. These features were suggestive, but not confirmatory, of varices or vascular malformation. A neurosurgery consultation suggested observation for the orbital bony defect.

**Figure 2a:**
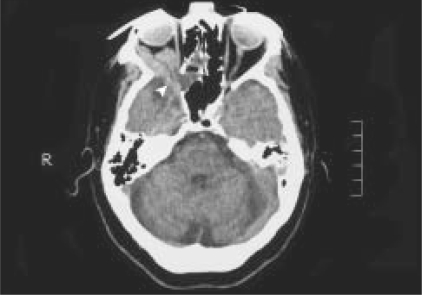
Computed tomographic scan showing absence of the greater wing of the sphenoid on the right side (arrowhead) and a defective right medial wall

**Figure 2b:**
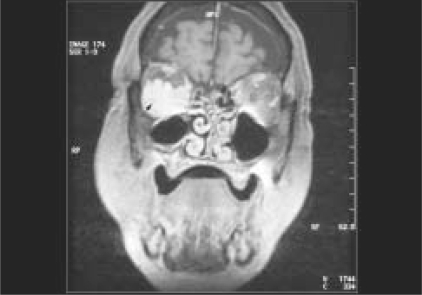
T2-weighted MRI scan reveals an intensely enhancing irregular mass lesion in the inferior orbit within which several flow voids can be observed (arrow)

As the clinical features and imaging were unable to provide a definitive diagnosis, an exploratory inferior orbitotomy was undertaken. Upon induction of general anesthesia, the right eye became obviously proptotic [Fig. 1b], but could easily be pushed back into the orbit, indicative of a compressible orbital mass. The orbitotomy was performed via a swinging eyelid, inferior transconjunctival approach. On incising the inferior orbital septum, an irregular mass of thin distensible vessels filled with blood came into view [Fig. 1c]. This appearance was typical for orbital varices and no biopsy was performed. The patient made an uneventful recovery.

## Discussion

Enophthalmos has been reported in association with orbital varix and is attributed to orbital fat atrophy.[4] Pulsating enophthalmos is a rare condition and is almost invariably associated with defects in the orbital bony wall, which allows transmission of brain pulsation. It is most commonly seen with defects of the greater wing of the sphenoid in patients with neurofibromatosis Type I.[5] It can rarely occur following extensive cranio-orbital bone resection.[6] In the present case, pulsating enophthalmos was secondary to defects in the medial orbital wall and greater wing of the sphenoid which were associated with an orbital varix. The association between cranial bony defects, encephaloceles and orbital varices has recently been reported.[2] Islam *et al.* found that orbital varices were associated with three types of cranial anomalies: major midline encephalocele (Type 1); large superomedial defects of the orbital wall (Type 2); and defects of the greater wing of the sphenoid (Type 3). According to this classification, our case appears to have a combination of Type 2 and Type 3 anomalies (defects in medial wall and greater wing of sphenoid). The bony defects have been attributed to a local failure of craniofacial development.[2]

It is interesting to note that Menon *et al.* reported a 14-year-old male with pulsating enophthalmos and isolated sphenoid wing aplasia.[7] The authors noted an orbital mass which increased in size with Valsalva maneuver but did not comment further on the mass. Review of their paper suggests that this was an orbital varix associated with a bony orbital defect.

Orbital varices have been divided into distensible and non-distensible types but both are known to have communications with the systemic venous circulation.[8] The proptosis of the right eye in our patient on induction of general anesthesia could only have been due to distension of the varices. Extubation-induced distension of an orbital varix has been reported[9] and induction with positive pressure ventilation is known to significantly raise the extrathoracic venous pressure.[10] The absence of distension with Valsalva maneuver in our case suggests a small communication between the varices and the orbital venous bed. This would have made the transmission of pressure difficult during positional change or Valsalva maneuver, but possible with positive pressure ventilation.

Orbital varices are usually managed conservatively since excision carries a small risk of significant hemorrhage. However, partial or complete excision can be performed for functional or cosmetic reasons. The use of hypotensive anesthesia and intra-operative injection (under direct visualization) of glue or other sclerosing agents helps in controlling bleeding and aids excision.[8]

## Conclusion

In summary, our case adds to the literature on the association between orbital varices and bony defects and illustrates the following points: it is important to image all patients with clinically suspected orbital varices as they may be associated with multiple cranial bony defects and encephaloceles; increase or induction of proptosis with general anesthesia and positive pressure ventilation should suggest an orbital varix even in the absence of distension with Valsalva maneuver clinically.
